# Efficacy of abdominal ultrasound inspection in the diagnosis and prognosis of neonatal necrotizing enterocolitis

**DOI:** 10.6061/clinics/2021/e1816

**Published:** 2021-03-19

**Authors:** Hong-Xia Gao, Bin Yi, Bao-Hong Mao, Wei-Yang Li, Xiang Bai, Yue Zhang, Jian-Ming Tang, Pei-Qi Liu, Kun Cheng

**Affiliations:** I Department of Neonatology, Gansu Provincial Maternity and Child Care Hospital, Lanzhou, 730050, China.; II Department of Clinical Medical Research Center, Gansu Provincial Maternity and Child Care Hospital, Lanzhou, 730050, China.; III Department of Neonatology, Capital Healthcare Hospital for Children and Women, Beijing, 102600, China.

**Keywords:** Necrotizing Enterocolitis, Abdominal Ultrasound, X-ray, Neonate, Diagnosis

## Abstract

**OBJECTIVE:**

This study aimed to identify the most useful ultrasound (US) features associated with definite neonatal necrotizing enterocolitis (NEC) and their prognostic values, particularly the calculated markers combined with important features.

**METHODS:**

A total of 213 suspected NEC cases were collected from the neonatal department of our hospital from January 2015 to August 2017. Each infant received both X-ray and US examinations.

**RESULTS:**

No differences were found in sex composition and delivery modes between groups. NEC-positive neonates had poorer prognosis compared to negative ones. The NEC group showed a higher frequency of abnormal signals. US showed higher NEC-related frequencies in different parameters. A variable (named predictor in US [PUS]) with five features was constructed. For NEC diagnosis, this variable provided a much higher area under the curve Q2 (AUC) (0.965) than other parameters. In this model, PUS had a cutoff value of 0.376 with a 0.900 sensitivity and 0.922 specificity. In prognosis, the closest factors were selected to draw a receiver operating characteristic curve, as well as a novel calculated variable US prognostic (USPro) marker. USPro had a much higher AUC (0.86) than other single features and showed a cutoff value of 0.18145, with 0.75 sensitivity and 0.84 specificity. This variable had a weaker power in prognosis when compared with PUS in diagnosis.

**CONCLUSIONS:**

The application of abdominal color Doppler US can provide high accuracy and sensitivity in NEC diagnosis and also contribute to its prognosis, without induction of radiation. Suspected neonates should be examined using this technique as early as possible.

## INTRODUCTION

Necrotizing enterocolitis (NEC) is a severe gastrointestinal disease that occurs during the developmental period. It affects 5-14% of infants weighing <1,500g (1). Among common neonatal gastrointestinal urgencies, NEC is highly life-threatening, with a high mortality if not treated in due course (2-6). So far, the etiology of NEC is still multifactorial (7,8). In pathological morphology, decreased perfusion and ischemia of the intestinal wall may cause disruption of the intestinal barrier, which enables bacterial infection and inflammation (9-15). It is crucial for suspected neonates to receive diagnostics and appropriate treatment as early as possible. Generally, the pediatrician may suspect NEC based on the typical and suggestive symptoms such as abdominal bloating, agitation, gastrointestinal residue retention, vomiting, and gastrointestinal bleeding. Apart from symptom observation, imaging findings play an essential role in the early diagnosis of NEC and initiation of prompt therapy.

Plain abdominal X-ray is currently considered the modality for the diagnostic protocol (16). However, radiographic evaluation often yields nonspecific findings and exposes the infants to radioactivity. Also, radiographs remain unfavorable for interpretation, because of its huge variability and serious overlap between signs of NEC and other intestinal pathologies. In our clinical practice, we noticed that abdominal ultrasound (US) examination might be helpful in the diagnosis of NEC. Theoretically, US visualizes the intestinal walls, gastrointestinal lumen, and neighboring structures; it depicts real-time images of the bowel and fluid in the peritoneal cavity, which collectively provide NEC features that cannot be seen with radiograph (*e.g.*, peristalsis and presence or absence of bowel wall perfusion) (17). When fully utilized, US could possibly behave more sensitively to detect intramural gas, portal venous gas (PVG), and free gas; it provides an opportunity to capture images of the bowel loops in cross section with dynamic evaluation (18). Based on US, the range of morphological changes is largely expanded. Moreover, Doppler US examination has the advantage of non-/low radiation, simple operation, and wide availability. Therefore, the primary objective was to enhance the advantages of abdominal color Doppler US inspection in NEC diagnosis and prognosis, especially in comparison with X-ray imaging. Additionally, US features may have a high specificity but relatively low sensitivity as some scholars mentioned (19-21). We hypothesized that any feature alone is limited in NEC recognition and outcome prediction, but a combination of those factors can enhance the efficacy. In summary, the objectives of this study were (1) to identify the US features associated with NEC and (2) to investigate the potential prognostic value of abdominal US in NEC.

## METHODS

### Patients

This retrospective study was approved by the Research Ethics Committee of Gansu Provincial Maternity and Child Care Hospital (no. 2014-12). The sample size was designed to be at least around 100 cases in each group. Available data were collected from the Department of Neonatology, Gansu Provincial Maternity and Child Care Hospital, Lanzhou, from January 2015 to August 2017. General demographic and clinical profiles such as birth weight, gestational age, time of starting milk, blood test abnormalities, NEC appearance time, when NEC was suspected, and routine blood test data were recorded in medical records. These data were correctly recorded and later compared between two groups. If the gestational age, sex distribution, or delivery mode were different between groups, they were matched after some outlier values were deleted, to ensure that baselines of the above parameters were comparable. Besides, the Apgar Score System was assessed at 1 min and 5 min postnatal. Each infant received both X-ray and US imaging.

The inclusion criteria were as follows: gestational age ≤36 weeks, body weight >1 kg, and age ≤7 days. The exhibition met the standard of Bell grading NEC performance (phase I). The exclusion criteria were as follows: (1) bloating symptoms that disappeared within 12h, (2) severe sepsis, (3) intestinal malformations, (4) severe persistent pulmonary hypertension of the newborn, and (5) those who abandoned treatment.

NEC diagnosis was achieved by two experts relying on a combination of clinical symptoms and radiological features. In detail, the clinical manifestations included feeding intolerance, abdominal distension, bloody stools, and/or sepsis; the laboratory findings included abnormal blood picture, metabolic acidosis, electrolyte disturbance (especially hyponatremia), positive stool occult blood, and/or positive blood and stool culture; the imaging features included intestinal dilatation, intestinal loops fixed, intestinal wall thickening, gas accumulation between the intestines, gas accumulation in the portal vein, and intestinal perforation in severe cases.

According to Bell’s NEC classification standard, the optimal treatment for NEC is fasting (3-5 days for mild cases, 7-14 days for grade II and above) and antibiotic therapy (ceftezole and sulbactam sodium as the first-line drug, imipenem as the next choice if infection control is not satisfactory, and meropenem is used for intracranial infection). When the clinical performance improved and the laboratory infection indicators returned to normal, antibiotics were discontinued, while intravenous nutrient treatment was given until the patient was discharged. The patients were recommended surgery and abdominal drainage if necessary.

Additionally, the results were documented as categorical data: 1, cured (receiving complete enteral nutrition, with normal weight gain and good general condition) 2, improved (receiving complete parenteral nutrition or beginning to receive enteral nutrition, with normal weight gain and good general condition); 3, deteriorated (receiving complete parenteral nutrition, with poor general condition, poor or no weight gain, or increased ascites, severe peritonitis, or intestinal perforation).

### X-ray and US imaging

X-ray and US imaging were performed as early as possible when a baby was suspected with NEC, and all data were collected within 24h of birth. Only images of the first detection were used for the analysis in this study. To avoid any potential bias, the US examiner and the X-ray radiologists were separate physicians. The Siemens^®^ Mobilett XP Digital DR X-ray Imaging System (Siemens Aktiengesellschaft, Munich, Germany) was used for X-ray film acquisition. The baby was placed in the supine position, and the abdomen was imaged anteriorly and posteriorly, and abdominal upright and horizontal projection images were documented. Simultaneously, Doppler sonographic examination was performed and read by the same experienced sonographer using the Philips^®^ HD11XE color Doppler US system (Koninklijke Philips N.V. Amsterdam, The Netherlands) (10- to 13-MHz high-frequency line array). Similarly, the baby was placed in the supine position, and the abdomen was projected anteriorly and posteriorly. The probe surface was covered with a coupling agent and then wrapped with disposable sterile gloves. The observation focus was the bowel wall echo and movement. X-ray and US imaging were performed at the same time point (the interval between the two examination did not exceed 1h). If some examinations were performed more than once (at different time points), only data of the first examination were analyzed.

The standards of key X-ray features were as follows:

- Intramural gas: Line or ring translucent shadow, bubble-like, along the intestinal wall, or blurred intestinal wall.
- PVG: Intrahepatic dendritic translucency.
- Bowel dilatation (BD): Bowel width greater than the lumbar spine diameter.
- Reduced inflation: No translucent shadow in part or all of the small intestine.
- Pneumoperitoneum: In the supine position, there is a triangular translucent shadow under the front abdominal wall.

The standards of key US features were as follows:

- Intramural gas: Granular, stripe, ring-shaped hyperechoic in the intestinal wall.
- PVG: Movable granular hyperechoic within the portal vein and its branches.
- Reduced inflation: Reduced or no hyperechoic gas probed in the small intestine.
- Decreased intestinal peristalsis: Intestinal peristalsis less than 10 times/min.
- Undetectable intestinal peristalsis: Intestinal motility is not detected.
- Seroperitoneum: Detectable free liquid dark areas in the abdominal cavity.

**Figure 1:**
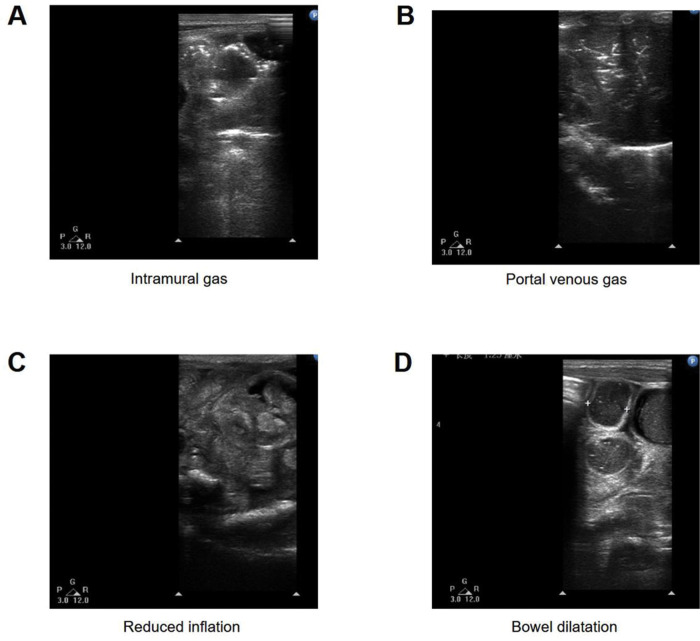
Typical features of US: (A) intramural gas, (B) portal venous gas, (C) reduced inflation, (D) bowel dilatation.

Several typical features of US are presented in Figure 1.

### Statistical Analysis

Data were described as mean±standard deviation and frequencies or percentages. Statistical calculations were conducted using SPSS 23.0 (International Business Machines Corporation, Chicago, USA), and GraphPad Prism was used for data visualization. Student’s t-test was conducted to compare numerical variables of independent samples between two groups (NEC *vs.* normal) when they were normally distributed, or else the Mann-Whitney U test was applied. The chi-squared test was performed to compare the categorical data. Receiver operating characteristic (ROC) curves of the selected variables were generated using SPSS. A *p*-value<0.05 was considered to be statistically significant.

## RESULTS

### Patient characteristics

A total of 213 suspected NEC cases were used for the analysis, including 102 negative (normal) and 111 definite NEC cases. The clinical characteristics of the two groups are shown in Table 1. No differences were found in the sex composition and delivery modes between groups, and the Apgar scores at 1 min and 5 min were comparable. When the prognosis was divided into the cured/improved/deteriorate categories, the NEC neonates had poorer prognosis compared to the negative controls (*p*<0.0001). According to abnormal signals, the NEC group showed a higher frequency of abnormal findings in both X-ray and US detection (X-ray, *p*=0.02; US, *p*<0.0001).

### X-ray and US features of NEC

The X-ray features of NEC are listed in Table 2. The NEC group had significantly increased frequencies of intramural gas (*p*<0.0001) and PVG (*p*<0.0001) and significantly decreased inflation reduction (*p*<0.05). In comparison, US provided more useful parameters with statistical significances (Table 3). The NEC group exhibited higher frequencies in intramural gas (*p*<0.0001), PVG (*p*<0.0001), BD (*p*<0.0001), decreased intestinal peristalsis (*p*<0.001), and undetectable intestinal peristalsis (*p*<0.0001), as well as less inflation reduction (*p*<0.05). Besides, NEC neonates had significantly higher intestinal wall thickness (*p*<0.0001) and thickness increase (*p*<0.0001). The US technique provided more differential dimensions compared to the X-ray imaging. Moreover, some differential features in the US method showed a higher positive frequency than X-ray: intramural gas (64 *vs.* 27), PVG (79 *vs.* 20), BD (85 *vs.* 34), and inflation (14 *vs.* 11).

### ROC curve of important parameters in X-ray and US

**Figure 2:**
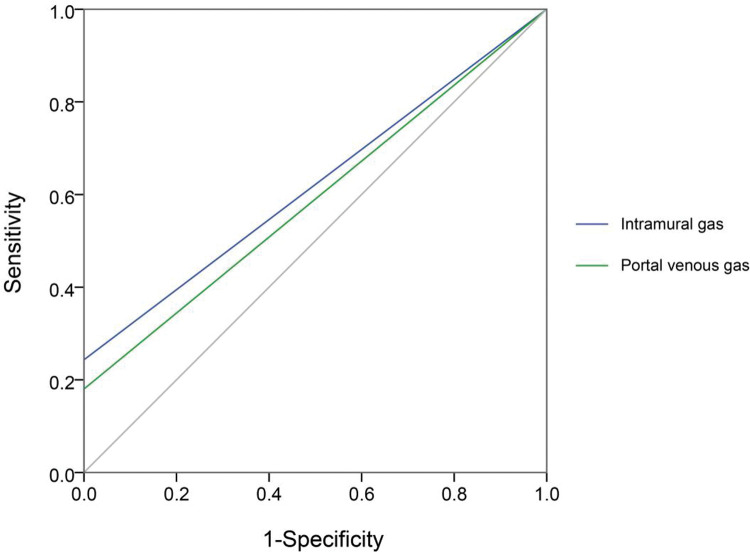
The receiver operating characteristic curve of two important variables in X-ray films (intramural gas and portal venous gas) in definite necrotizing enterocolitis recognition.

**Figure 3:**
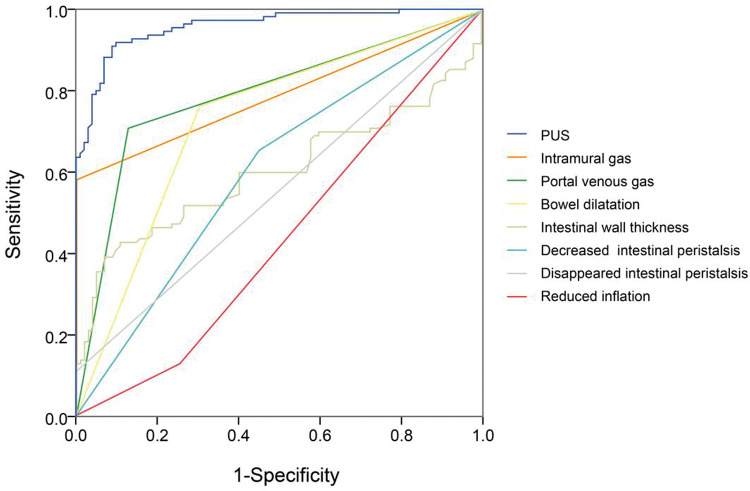
ROC of seven variables of US features in NEC diagnosis, including intramural gas, portal venous gas, intestinal wall thickness, bowel dilatation, reduced inflation, decreased intestinal peristalsis, and undetectable intestinal peristalsis. Besides, we constructed a variable (named predictor in US [PUS]) with five features (intramural gas, portal venous gas, intestinal wall thickness, bowel dilatation, and decreased intestinal peristalsis; intestinal wall thickness was a numerical variable expressed in millimeter, and the other four variables were categorical data that were valued 0 or 1, with 0=no and 1=yes). Relatively, this predictor can provide a much higher AUC (0.958) than the other parameters. In this model, PUS had a cutoff value of 5.44 with a sensitivity of 0.92 and a specificity of 0.90.

Next, we compared each diagnostic parameter using the ROC curve. Two variables in X-ray films (intramural gas and PVG) (Figure 2) and seven US features (intramural gas, PVG, intestinal wall thickness, intestinal wall thickness increase, BD, decreased inflation, decreased intestinal peristalsis, and undetectable intestinal peristalsis) (Figure 3) had significant diagnostic values. Further, these factors were used in a linear regression for NEC diagnosis. The model was established by SPSS, and the regression formula was modified to generate a more concise and easily calculable variable (named predictor in US [PUS]). It was constructed with five features using the following formula: 5*IG+3*PVG+2*IWT+2*BD+1.5*DeIP+DiIP. (Here, IG=intramural gas, PVG=portal venous gas, IWT=intestinal wall thickness, BD=bowel dilatation, RI=reduced inflation, DeIP=decreased intestinal peristalsis, and DiIP=undetectable intestinal peristalsis. IWT was a numerical variable expressed in millimeter, and the other four variables were categorical data that valued 0 or 1, with 0=no and 1=yes; Figure 3.) This predictor provided a much higher AUC (0.958) than the other parameters in NEC recognition. In this model, PUS had a cutoff value of 5.44 with a 0.92 sensitivity and 0.90 specificity.

### Prognostic values of the US features

**Figure 4:**
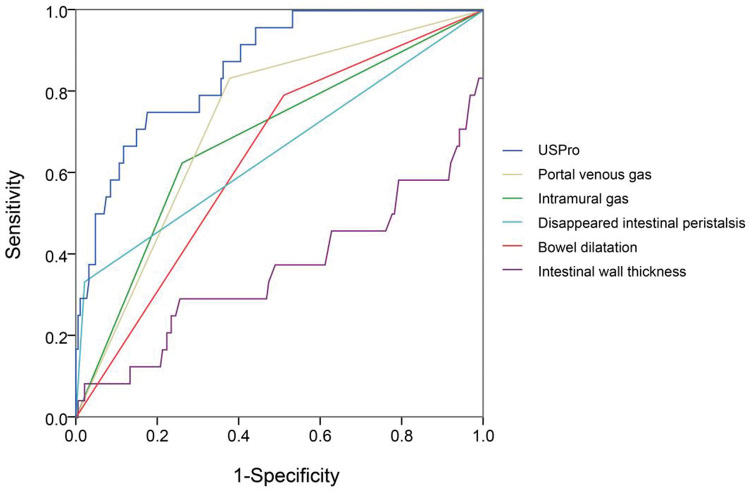
ROC of important US features, including intramural gas, portal venous gas, thickness increase, thickness increase, bowel dilatation, reduced inflation, and undetectable intestinal peristalsis. The associated factors were selected to calculate a novel variable US prognostic (USPro; intramural gas, portal venous gas, intestinal wall thickness, thickness increase, bowel dilatation, reduced inflation, decreased intestinal peristalsis, undetectable intestinal peristalsis, intestinal wall thickness, and thickness increase were numerical variables expressed in millimeter, and the other variables were categorical data that were valued 0 or 1, with 0=no and 1=yes). USPro had a much higher AUC (0.861) than other single features. To predict the third outcome (deteriorated), USPro showed a cutoff value of 0.96, with a sensitivity of 0.67 and a specificity of 0.88.

In view of the superiority of US imaging in NEC diagnosis, we further explored the correlation between US features and the outcomes. The outcomes were documented as follows: cured (coded 1), improved (coded 2), and deteriorated (coded 3). As shown in Table 4, the Pearson R correlation between US features and outcomes was calculated, in which a higher R means a poorer outcome (or a higher possibility of deterioration). The outcome was significantly correlated with intramural gas, PVG, intestinal wall thickness, BD, reduced inflation, and undetectable intestinal peristalsis. Similarly, the key factors were selected to draw a ROC curve, as well as a novel constructed variable USPro marker: USpro=3*IG+6*PVG-14*IWT+3*BD+2*RI+DeIP+20*DiIP. (Here, IG=intramural gas, PVG=portal venous gas, IWT=intestinal wall thickness, BD=bowel dilatation, RI=reduced inflation, DeIP=decreased intestinal peristalsis, and DiIP=undetectable intestinal peristalsis, IWT was a numerical variable expressed in millimeter, and the other variables were categorical data that valued 0 or 1, with 0=no and 1=yes.) As shown in Figure 4, USPro had a much higher AUC (0.861) than other single features. To predict the third outcome (deterioration), USPro provided a sensitivity of 0.67 and a specificity of 0.88 at the cutoff value of 0.96. As shown in the ROC curve, the efficacy of this variable is satisfactory, but the power is weaker in comparison with PUS in diagnosis. Collectively, US features had a high diagnostic value and is also contributive in NEC prognosis.

## DISCUSSION

In this work, we compared the efficacy between US and X-ray imaging in neonatal NEC diagnosis, and we observed that US showed a significant priority. In comparison with known facts, the strengths of this study are as follows: (1) We revealed that the US technique provides not only more differential parameters compared to X-ray, but also a closer correlation between these markers and NEC; (2) when using the novel marker PUS as we proposed, the NEC can be distinguished by a ROC with AUC around 0.958, and the PUS threshold 5.44 can be applied in NEC alert (sensitivity=0.92, specificity=0.90). In addition, the calculated marker USPro with 0.96 threshold can be regarded as a warning of poor prognosis. Our work strongly suggests that abdominal color Doppler US can be applied as early as possible in the diagnosis of NEC and that the combined application of X-ray and US protocols can improve the accuracy and sensitivity. In the future, using the predictive factors we provided, neonatologists can predict NEC as early as possible and prevent its development in a timely manner.

So far, previous studies have analyzed the usefulness of US in neonatal NEC evaluation (22), and the evaluation of the bowel by US has been shown to have high specificity for detecting bowel necrosis (30). In 2013, a case report showed that bowel distension and intraluminal gas were frequently detected in NEC neonates, and bowel wall thickening together with increased bowel wall perfusion was observed in nine patients. This work suggested that US examination can be helpful in the initial diagnosis as well as the follow-up of patients developing NEC (23). Another study reported that PVG, BD, and bowel wall thickening frequencies in the NEC group were higher in the US protocol than in plain X-rays (24), which is consistent with our results and suggests a diagnostic value and disease evaluation significance of abdominal US inspection for neonatal NEC. Similarly, US outperforms abdominal X-ray plain film in the detection rates of PVG, dilatation of the intestine (27,28), intramural air, and so on. In 2018, a meta-analysis by Chan et al. systematically reviewed the works about the diagnostic accuracy of US in the diagnosis of NEC (29). They discovered that individual signs of NEC (PVG, pneumatosis, free air, bowel wall thinning, absent peristalsis, and abdominal fluid) had overall low sensitivities and high specificities. These limitations may be addressed by our model with combined variables (PUS, Figure 2). Collectively, it has been repeatedly accepted that abdominal US should become part of the standard care for early NEC diagnosis (31).

Besides diagnosis, known studies also suggested the prognostic significance of US (17,31). Yuldashev et al. have assessed the prognostic value of abdominal color Doppler US in NEC development (25). Also, complex fluid collection with abdominal US was reported to be correlated with the need for surgery in newborn infants with severe NEC (26). Chan et al. conducted a meta-analysis to identify bowel US findings associated with surgical management or death in infants with NEC (20), and they revealed that focal fluid collections, complex ascites, absent peristalsis, pneumoperitoneum, bowel wall echogenicity, bowel wall thinning, absent perfusion, bowel wall thickening, and BD were associated with surgery or death in NEC patients. This is partially in line with our novel marker USPro, despite the power of our marker that is still to be improved. Besides, some of the above US features (which can be commonly observed in radiographs) were confirmed constructive in prognosis in combination with radiological findings. Together, US examination allows for more accurate assessment of NEC onset and possible outcomes (32) and helps clinicians in making more accurate therapeutic decisions. Although abdominal radiographs may remain the gold standard for NEC diagnosis because radiologists are familiar with the features of radiographs, we believe US inspection may gradually be acknowledged as superior.

Additionally, there were some positive X-ray and US features in the normal group. For example, there were 13 PVG cases, 31 BD cases, and 9 seroperitoneum cases in the normal group (Table 3). The possible reasons are as follows: (1) There are indeed some features that seemed abnormal but actually occur in normal neonates. Single features mentioned above do not necessarily have a diagnostic value. (2) The infants enrolled were all suspected NEC cases but not purely healthy ones, and these features may be early manifestations of NEC development but without sufficient powers in distinguishing them.

The limitations of this study are the following. First, we have not observed factors influencing the prognosis in detail, and the prognostic marker USPro has a relatively weaker power in comparison with PUS. Due to the lack of follow-up data, the usefulness (as indicated by the ROC curve) of USPro is moderate. More information about the treatment, the detailed responses to therapies, and the growth and development after discharge are to be collected. Also, abdominal color Doppler US should be performed for the baby, and the time point should be after birth; whether it has a predictive role before delivery is still unclear. Moreover, the diagnostic role of US may be improved in combination with detailed examination of signs and symptoms, which has not been investigated in our study.

## CONCLUSIONS

The application of abdominal color Doppler US can provide high accuracy and sensitivity in NEC diagnosis, which also contributes to its prognosis, without induction of radiation. Suspected neonates should be examined with this technique as early as possible.

## AUTHOR CONTRIBUTIONS

Gao HX participated in the design of this study, and all of the other authors performed the statistical analysis. Yi B, Mao BH, Li WY, Bai X, Zhang Y, Tang JM, Liu PQ and Cheng K carried out the study and collected important background information. Gao HX drafted the manuscript. All of the authors read and approved the final version of the manuscript.

## Figures and Tables

**Table 1:** Clinical characteristics of 213 neonates.

Parameter		Normal (%)		NEC (%)		*p-*value
Sex	Male	47	46%	62	56%	0.171
	Female	55	54%	49	44%	
Delivery mode	Eutocia	45	44%	51	46%	0.367
	Cesarean section	57	56%	58	52%	
	Others	0	0%	2	2%	
Gestational age (week)		33.54±1.81		33.27±2.08		0.26
Birth weight (KG)		18.41±0.41		16.78±0.39		<0.0001
Prognosis	Cured	97	95%	83	75%	<0.0001
	Improved	3	3%	6	5%	
	Deteriorated	2	2%	22	20%	
Abnormal X-ray signals	Yes	60	59%	82	80%	0.02*
	No	42	41%	29	28%	
Abnormal US signals	Yes	88	86%	111	100%	<0.0001
	No	14	14%	0	0%	
Apgar score at 1 min		8.10±1.87		7.90±1.59		0.405
Apgar score at 5 min		8.95±2.00		8.83±1.99		0.656

**Table 2:** X-ray features of NEC.

Features		Normal	NEC	*p-*value
Intramural gas	No	102/0	84/27	<0.0001
	Yes			
Portal venous gas	No	102/0	91/20	<0.0001
	Yes			
Bowel dilatation	No	80/22	77/34	0.133
	Yes			
Reduced inflation	No	79/23	100/11	0.012
	Yes			
Pneumoperitoneum	No	102/0	110/1	0.337
	Yes			

**Table 3:** US features of NEC.

Features		Normal	NEC	*p*-value
Intramural gas	No	102	47	<0.0001
	Yes	0	64	
Portal venous gas	No	89	32	<0.0001
	Yes	13	79	
Intestinal wall thickness (mm)		0.75±0.14	0.89±0.31	<0.0001
Bowel dilatation	No	71	26	<0.0001
	Yes	31	85	
Reduced inflation	No	76	97	0.016
	Yes	26	14	
Decreased intestinal peristalsis	No	56	39	0.003
	Yes	46	72	
Undetectable intestinal peristalsis	No	102	98	<0.0001
	Yes	0	13	
Seroperitoneum (no/yes)	No	93	100	0.786
	Yes	9	11	

**Table 4:** Correlation between US features and outcomes.

Features	Correlation (R)	*p*-value
Intramural gas	0.249	<0.0001
Portal venous gas	0.316	<0.0001
Intestinal wall thickness	-0.127	0.011
Bowel dilatation	0.190	0.001
Reduced inflation	-0.014	0.026
Decreased intestinal peristalsis	-0.054	0.340
Undetectable intestinal peristalsis	0.401	<0.0001
